# Hyper-reflective retinal foci as possible *in vivo* imaging biomarker of microglia activation in von Hippel-Lindau disease

**DOI:** 10.1371/journal.pone.0272318

**Published:** 2022-08-12

**Authors:** Elisabetta Pilotto, Tommaso Torresin, Maria Laura Bacelle, Gilda De Mojà, Alfonso Massimiliano Ferrara, Stefania Zovato, Giulia Midena, Edoardo Midena

**Affiliations:** 1 Department of Neuroscience—Ophthalmology, University of Padova, Padova, Italy; 2 Oftalmico Hospital, ASST Fatebenefratelli Sacco, Milano, Italy; 3 Familial Tumor Unit, Veneto Institute of Oncology IOV-IRCCS, Padova, Padova, Italy; 4 IRCCS, Fondazione Bietti, Rome, Italy; UPMC and Pittsburgh School of Medicine, UNITED STATES

## Abstract

**Purpose:**

von Hippel-Lindau (VHL) disease is caused by a mutation of the VHL gene and characterized by the development of retinal hemangioblastomas (RH). Current pathophysiologic mechanisms of RH development and progression are still insufficient to predict RH behavior. VHL gene is involved in the cellular response to hypoxia and in many intracellular signaling pathways expressed both in angiogenesis and inflammation. Optical coherence tomography (OCT) allows to identify hyper-reflective retinal foci (HRF) known as aggregates of activated microglial cells as possible *in vivo* biomarker of local inflammation. The aim of the present study was to investigate the presence of HRF in patients with genetically confirmed VHL disease.

**Methods:**

In this cross-sectional study, patients with VHL underwent complete ophthalmological examination and OCT with HRA + OCT Spectralis. HRF were manually identified and calculated in inner (IR), outer (OR) and full retina. Age-matched healthy subjects were enrolled as controls.

**Results:**

113 eyes of 63 VHL patients and 56 eyes of 28 healthy subjects were evaluated. HRF number was significantly higher in VHL than in controls in IR (28.06 ± 7.50 vs 25.25 ± 6.64, p = 0.042). No difference was observed in OR and in full retina (OR: 7.73 ± 2.59 vs 7.95 ± 2.51, p = 0.599; full retina: 35.79 ± 8.77 vs 33.20 ± 7.47, p = 0.093).

**Conclusion:**

The increase of HRF, which mirror retinal microglial activation, characterizes VHL eyes. The role of activated microglia in the retina of VHL eyes needs to be better investigated, mainly considering local VHL disease manifestations.

## Introduction

Von Hippel- Lindau disease is an autosomal dominantly inherited cancer syndrome in which mutations in the tumor suppressor VHL gene is believed to cause the development of characteristic tumors in the central nervous system, the eye and internal organs [1]. Retinal hemangioblastoma (RH), one of the earliest and most frequent manifestations of VHL disease, originates in the neurosensory retina or optic disc area, and is composed of VHL-inactivated foamy stromal cells and abundant reactive vessels [1, 2]. Vision loss is usually caused by intraretinal exudation involving the macula secondary to RH or by vitreomacular traction and preretinal fibrosis leading to exudative and/or tractional retinal detachment [3]. The response to treatment of RH (laser photocoagulation, cryotherapy, photodynamic therapy, brachytherapy and, more recently, intravitreal anti-VEGF drugs or systemic propranolol) is still unpredictable [4, 5]. This means that current pathophysiologic mechanisms of RH development and progression are still insufficient to predict RH behavior, even when sophisticated retinal imaging technologies are used. VHL gene is involved in many intracellular signaling pathways expressed both in angiogenesis and inflammation. In particular, VHL gene mutation disrupts the hypoxia-induced/vascular endothelial growth factor (VEGF) pathway and the NOTCH signaling. These altered pathways, as demonstrated in several systemic and retinal diseases, are essential for microglia activation. Indeed, retinal microglial cells are active sensors of the microenvironment, and microglial reactivity is a real hallmark of various diseases [6]. Spectral domain optical coherence tomography (OCT) allows the identification of solitary Hyper-reflective Retinal Foci (HRF). The main and widely accepted hypotheses refer HRF as aggregates of activated resident retinal microglial cells, playing a pivotal role in retinal inflammation [7–12]. An increased number of HRF was recently found after intraocular surgical procedure, and in patients suffering from Multiple Sclerosis, a typical neuroinflammatory disease, confirming HRF inflammatory nature [13–15].

The aim of this study was to evaluate the presence and amount of HRF in eyes of genetically confirmed VHL patients.

## Methods

### Participants

Patients suffering from genetically confirmed VHL disease who underwent annual ophthalmological evaluation at the Padova University Hospital between January 2020 and December 2020 were considered for this post-hoc analysis. Patients contributed with both eyes to the study. Exclusion criteria were: treated or untreated hemangioblastomas at the posterior pole or in the peripapillary area; macular retinal pigment epithelium mottling or macular scars; epiretinal membranes and macular pucker; previous or concomitant inflammatory eye diseases; previous or concomitant retinal vascular diseases; acquired or congenital anterior segment disorders; refractive defects greater than 6 D; intraocular surgery or any laser photocoagulation in the last six months. During the same period, healthy volunteers were also recruited as controls. The study followed the tenants of the Declaration of Helsinki. The approval from the Ethics Committee for Clinical Practice of the Padova University Hospital for the study was obtained (N° 59n/AO/20). Informed consent was obtained from each subject and data collection followed the tenets of the Declaration of Helsinki.

All subjects underwent complete eye examination including best corrected visual acuity (BCVA) measurement using ETDRS charts, anterior segment evaluation, intraocular pressure measurement, slit lamp biomicroscopy with 90D lens and indirect ophthalmoscopy with 20D lens after pupil dilation with 1% tropicamide. OCT examination was performed during the same day using HRA + OCT Spectralis Heidelberg (Heidelberg Engineering, Germany). OCT scans were acquired late in the morning, under mydriatic condition, in a dark room and with the in-built eye-tracker always activated. For the purpose of the study a single horizontal line scan (180° line scan, 9-mm length, with automated real time (ART) set at 100 frames) centred onto the fovea was acquired.

### Hyper-reflective retinal foci

**Fig 1:**
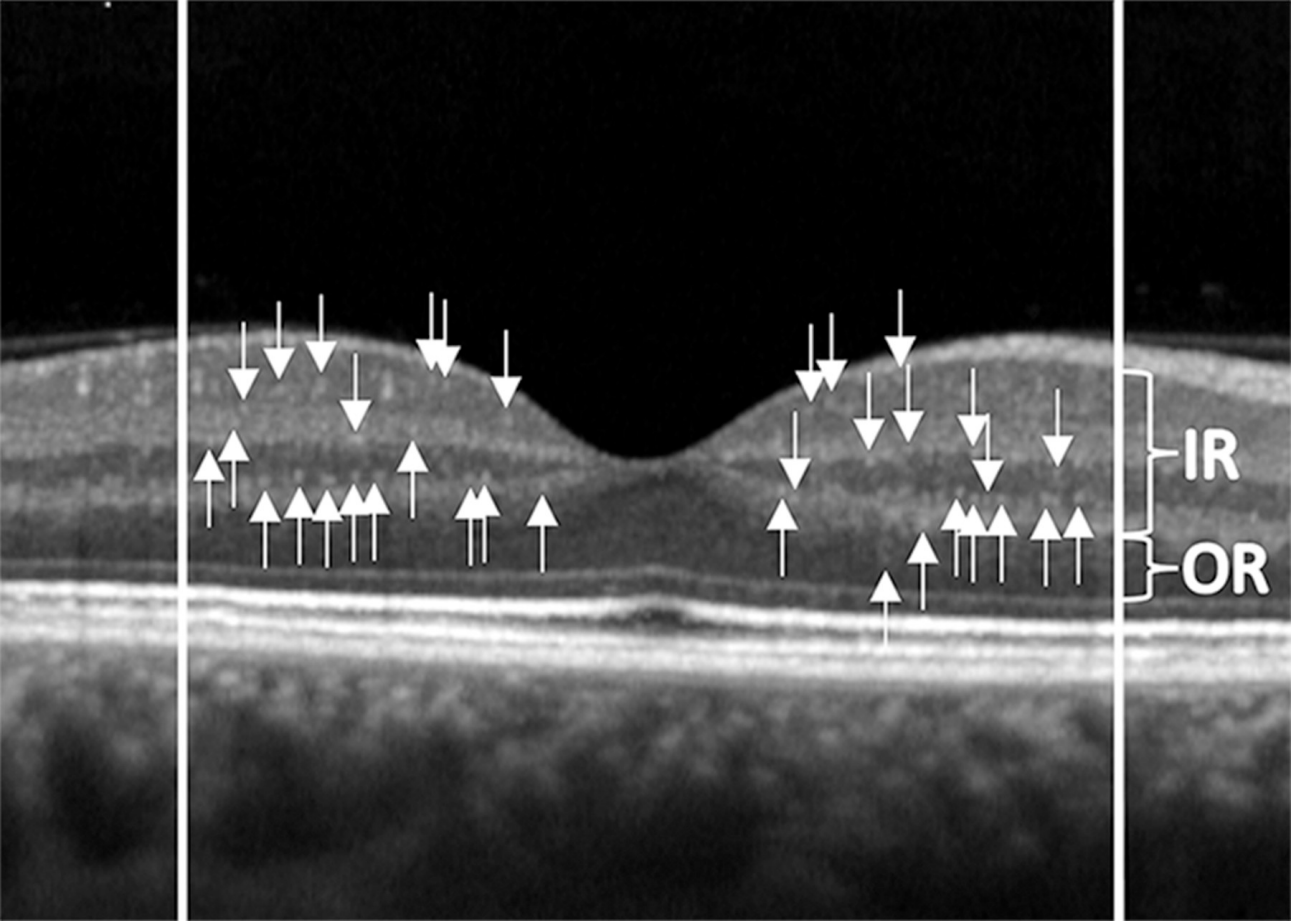
Hyperreflective retinal foci (HRF) and retinal segmentation. Hyperreflective retinal foci (HRF) at the OCT linear scan identified in the full retina, from the boundary between nerve fiber layer and ganglion cell layer (RNFL/GCL) to the external limiting membrane (ELM); in Inner Retina (IR), all layers between the boundary between RNFL/GCL and the lower limit of the outer plexiform layer; and in Outer Retina (OR), between the upper limit of the outer nuclear layer and the ELM.

As previously reported, HRF were defined as isolated punctiform elements of small dimensions (≤30μm) with intermediate reflectivity (similar to that of the nerve fiber layer) and without a shadow cone [16]. HRF were identified and counted in the central 3 mm, included between two perpendicular lines to Bruch Membrane traced at 1500 μm both temporally and nasally from the centre of the fovea [16]. HRF were identified and counted: in the full retina, from the boundary between nerve fiber layer and ganglion cell layer (RNFL/GCL) to the external limiting membrane (ELM); in Inner Retina (IR), all layers between the boundary between RNFL/GCL and the lower limit of the outer plexiform layer (OPL); and in Outer Retina (OR), between the upper limit of the outer nuclear layer (ONL) and the ELM. Two independent operators, that were blind to the clinical and demographic features, manually located (IR and/or OR) and counted HRF (TT and MLB) (Fig 1).

Each operator performed two assessments one week apart.

### Statistical analysis

All measures were described according to the usual methods of descriptive statistics: the qualitative parameters were expressed in terms of absolute and relative frequency (percentage), the quantitative ones in terms of arithmetic mean, standard deviation and interval of variation (minimum maximum). The Gaussian distribution of the parameters in the sample groups was verified by the Shapiro-Wilk test. Gender and age demography of both patients and controls were compared with Chi-square and t-Student tests for independent samples, respectively. The inferential analysis concerned HRF of the IR, OR and full retina. The mean values of the measured parameters were compared between the VHL patient group and controls using a one-factor ANOVA model (Group).

All models were adjusted for replication of measurements in the two eyes of the same subject.

Statistical analyzes were carried out using SAS® v.9.4 software (SAS Institute, Cary, NC, USA); statistical tests were interpreted as significant if p <0.05.

## Results

Sixty-three VHL patients (113 eyes) and 28 healthy individuals (56 eyes) were evaluated. All subjects were Caucasian and the two groups were homogeneous for gender distribution (p = 0.5496), mean age (p = 0.8846) and BCVA (p = 0.367) (Table 1).

**Table 1:** Demographic characteristics of enrolled VHL patients and healthy controls.

	VHL patients	Healthy Controls	*p* Value
Subjects, (number)	63	28	
Mean age (years: mean, SD)	39.6, 14.1	40.0, 11.8	0.8846
Female:Male (number, %)	34:29, 54:46	17:11, 60.7:39.3	0.5496
BCVA (letters: mean, SD)	85.30, 2.37	85.5, 1.12	0.3670

VHL: **von Hippel–Lindau** disease; SD: standard deviation; BCVA: best corrected visual acuity

Thirteen VHL patients contributed to the analysis with only one eye because of: RH at the posterior pole (5 eyes), band keratopathy (2 eyes), previous retinal detachment (1 eye), epiretinal membrane (3 eyes) juxtapapillary myelinated nerve fibers (1 eye) and macular retinal pigment epithelium mottling (1 eye) in the fellow eye. Therefore, 113 VHL eyes were analyzed. Forty-one eyes of 113 had peripheral RH: 23 eyes in zone 2 and 18 in zone 3. In 28 eyes, RH had been previously treated with laser photocoagulation.

**Fig 2:**
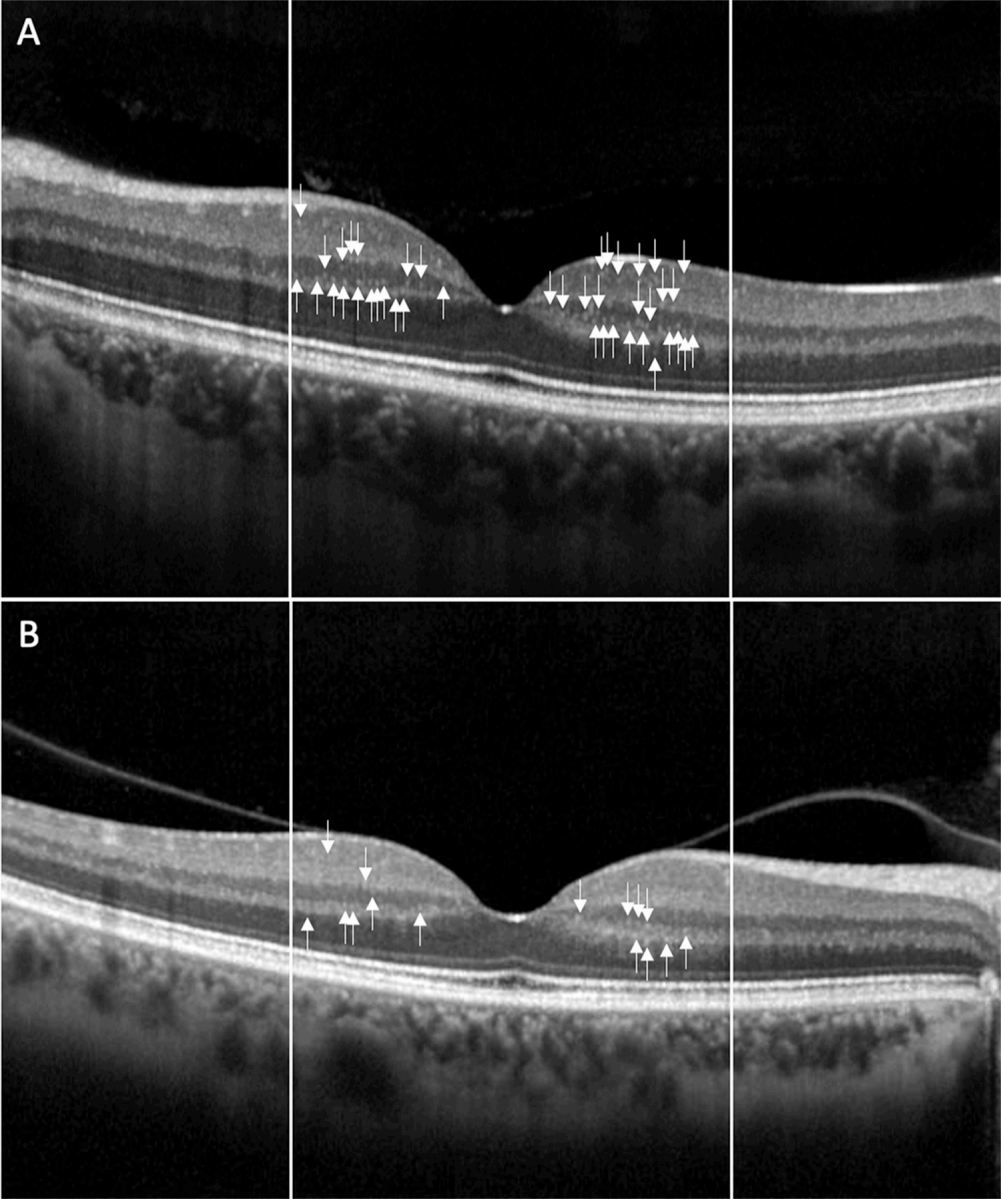
Example of hyperreflective retinal foci (HRF). Hyperreflective retinal foci (HRF) at the OCT linear scan in one VHL eye (A) and in one healthy control (B). HRF are more numerous in VHL eye than in control, mainly in the Inner Retina.

Mean HRF number in IR was significantly higher in VHL eyes compared to controls (p = 0.042) (Fig 2). Mean HRF number did not significantly differ between patients and controls in OR and in full retina (p = 0.599 in OR and p = 0.093 in full retina) (Table 2).

**Table 2:** Best corrected visual acuity and hyper-reflective retinal foci in VHL eyes compared to healthy controls.

	VHL eyes (113)	Healthy Controls (56)	*p* Value
HRF (numbers: mean, SD)			
Inner Retina	28.06, 7.50	25.25, 6.64	0.0420*
Outer Retina	7.73, 2.59	7.95, 2.51	0.5990
Full retina	35.79, 8.77	33.20, 7.47	0.0930

VHL: **von Hippel–Lindau** disease; HRF: hyper-reflective retinal foci; IR: inner retina; OR: outer retina; p value: statistically significant values marked (*)

No statistically significant difference in HRF was found between eyes with lesions and eyes without lesions in the inner retina (p = 0.3913), outer retina (p = 0.1567) or total retina (p = 0.2925). Also between treated and untreated eyes no significance difference was found in the inner retina (p = 0.4573), outer retina (p = 0.4133) or total retina (p = 0.6711).

Repeatability between the two operator who segmented the images was excellent with an intraclass correlation coefficient of 0.99 (95% confidence interval ranging from at least 0.98 to 0.99). Interobserver repeatability was with intraclass correlation coefficient of 0.82 (95% confidence interval: 0.71–0.99).

## Discussion

In this cross-sectional study, OCT imaging was used to quantify HRF in eyes of patients affected by genetically confirmed VHL disease. HRF, mostly considered aggregates of microglial cells, appear at structural OCT as intraretinal foci less then 30μm in size, with reflectivity similar to RNFL and without posterior shadowing [16]. Retinal microglia cells represent immune resident cells, mainly located in the inner retinal layers, which respond to various metabolic stresses with activation, accumulation and migration toward the outer retinal layers [9, 17–19]. Our analysis highlighted the presence of significantly higher number of HRF in the inner retina of VHL eyes. Even if the small difference in HRF is not currently clinically useful, this finding may provide additional knowledge in retinal involvement in VHL patients with or without RH. In VHL disease, the presence of an abnormal VHL protein (pVHL) prevents the correct degradation of hypoxia-inducible factors (HIFs), resulting in a cellular condition defined as "pseudo-hypoxia", with increase of VEGF [2, 20]. VEGF level in aqueous humor of VHL eyes is higher than in healthy subjects and is increased in stromal cells of RH [21, 22]. VEGF acts in the activation, proliferation and migration of microglial cells in the brain and in the retina in ocular inflammation [23, 24]. Moreover, VEGF receptor 1 has been detected in retinal microglia [25]. Peripheral and posterior pole perfusion impairment, recently described, may cause retinal tissue hypoxia [26–29]. In particular, in a previous study, we demonstrated with OCT angiography, that macular perfusion was reduced in VHL patients with and without RH, compared to controls, thus confirming the presence of an altered vascular environment [27]. Therefore, the VEGF overexpression, due to the mutation of pVHL, contributes to activation of microglia in VHL. Moreover, we hypothesize that pVHL alterations determine a “pre-activation” of the resident microglia and, according to Knudson’s two-hit hypothesis, the second hit in the retina occurs precisely in the proliferating glial cells [30]. Consequently, cellular “pseudo-hypoxia” and alterated vascular environment may justify the increase in HRF detected in VHL eyes.

HRF number was not increased in OR in VHL disease. Deficit of the pVHL influences also, the NOTCH signaling pathway [31]. Ligands and receptors of the NOTCH pathway are expressed in microglia, and NOTCH activation reduces the pro-inflammatory activity of microglial cells, as described in the central nervous system [31, 32]. In VHL disease, the NOTCH signaling pathway, which is hyper stimulated by the increased HIFs level, may prevent microglia migration towards the outer retinal layers. This could explain why HRF were not increased in the OR of VHL patients.

The main limitation of this study is the manual count of HRF on OCT scans which is time-consuming and requires expert and well-trained operators. New automatic or semi-automated methods have been recently described [33]. Another limitation is represented by the lack of genotype-phenotype correlations. Therefore, we cannot exclude that the genotype of VHL germline mutation may differently influence the microglia behavior in the retina [34–36].

In conclusion, in VHL eyes HRF number is increased in the inner retinal layers compared to healthy controls, hypothetically as a consequence of altered intracellular signaling pathways related to VHL gene mutation, which may induce retinal microglial activation. The clinical and pathogenetic significance of increased HRF in VHL disease deserve further investigations in prospective longitudinal studies, mainly considering intraocular VHL complications.
